# Insights into the role of CuO in the CO_2_ photoreduction process

**DOI:** 10.1038/s41598-018-36683-8

**Published:** 2019-02-04

**Authors:** André E. Nogueira, Jéssica A. Oliveira, Gelson T. S. T. da Silva, Caue Ribeiro

**Affiliations:** 1 Brazilian Nanotechnology National Laboratory (LNNano), Brazilian Center for Research in Energy and Materials (CNPEM), Zip Code 13083-970 Campinas, São Paulo Brazil; 2 Embrapa Instrumentation, Rua XV de Novembro, 1452, CEP: 13560-970, CP 741, São Carlos, SP Brazil; 3 Department of Chemical Engineering, Federal University of São Carlos, Via Washington Luiz, km 235, CEP: 13565-905 São Carlos, SP Brazil; 4 Department of Chemistry, Federal University of São Carlos, Via Washington Luiz, km 235, CEP: 13565-905 São Carlos, SP Brazil; 5 Forschungszentrum Jülich GmbH, Institut für Energie- und Klimaforschung IEK-3 Elektrochemische Verfahrenstechnik, 52425 Jülich, Germany

## Abstract

The CO_2_ photoreduction process to produce light hydrocarbons is known to be influenced by the presence of CuO nanoparticles, but the actual role of this material, whether as a catalyst or a reactant, has not yet been revealed. In this work, we investigate the role of CuO nanoparticles produced by a solvothermal method as a catalyst in CO_2_-saturated water reaction media under UV light, considering the effects of different electrolytes (Na_2_C_2_O_4_, KBrO_3_, and NaOH) and temperatures on nanoparticle phase and activity. The electrolyte strongly influenced product selectivity (NaOH led to evolution of CH_4_, Na_2_C_2_O_4_ to CO, and KBrO_3_ to O_2_) and induced CuO phase change. A long-term analysis of these processes indicated that during the initial steps, CuO acted as a reactant, rather than as a catalyst, and was converted to CuCO_3_.Cu(OH)_2_, while the as-converted material acted as a catalyst in CO_2_ photoreduction, with conversion values comparable to those reported in the literature.

## Introduction

The increase in use of fossil fuels for energy production raises serious concerns from the environmental point of view. Allied to this energy demand, the emission of carbon dioxide (CO_2_), the most significant gas related to the greenhouse effect, contributes significantly to climate change, requiring new strategic approaches and control of emissions^1,2^. In order to contribute to achieving sustainable sources of energy, photocatalytic materials have been developed for the conversion of CO_2_ to useful chemical compounds and fuels, employing solar ultraviolet (UV) and visible radiation in a so-called artificial photosynthesis process^3–5^.

In heterogeneous photocatalysis, when semiconductors are illuminated with energy equal to or greater than the energy of the band gap, electron transfer from the valence band (VB) to the conduction band (CB) generates electron/hole pairs, providing reductive and oxidative sites, respectively^6^. Photogenerated electrons in the conduction band can then react with molecules adsorbed on the material surface, such as CO_2_, which is reduced to carbon monoxide, methane, ethanol, formic acid, and other added-value chemicals^2,6–8^.

Understanding of the various stages of the process is fundamental for the development of materials with appropriate characteristics for this application, as well as to improve the reaction conditions by the elimination of interferents and the addition of species able to enhance the photoreduction efficiency. However, although high CO_2_ conversion values have been reported in the literature, the roles of different materials in this reaction were not revealed and there is no consensus concerning the most suitable material for catalysis of this reaction. The most studied semiconductor for this application is TiO_2_, however its low absorption in the visible region makes difficult the use of solar radiation in this process. Thus, a semiconductor that has been showing good results is copper oxide, but uncertainty remains about its actual role in the CO_2_ photoreduction, whether as a catalyst or as a reactant^9–12^. In addition, it is necessary to propose feasible photoreduction mechanisms and to determine the ways in which the most important reactive species influence product selectivity. To this end, evaluation of the effect of addition of electrolytes that act as radical, electron, or hole scavengers can clarify their roles in CO_2_ photoreduction^13–15^.

In this work, we systematically investigate CO_2_ photoreduction on CuO nanoparticles synthesized by a solvothermal method, employing different electrolytes and temperatures. The results revealed that CuO acted as a reactant, while as-formed copper carbonate could act as a catalyst in this reaction. The electrolytes influenced CuO phase change and product selectivity, helping to elucidate the ways in which the CO_2_ photoreduction process was assisted by this material.

## Results and Discussion

### Characterization

**Figure 1:**
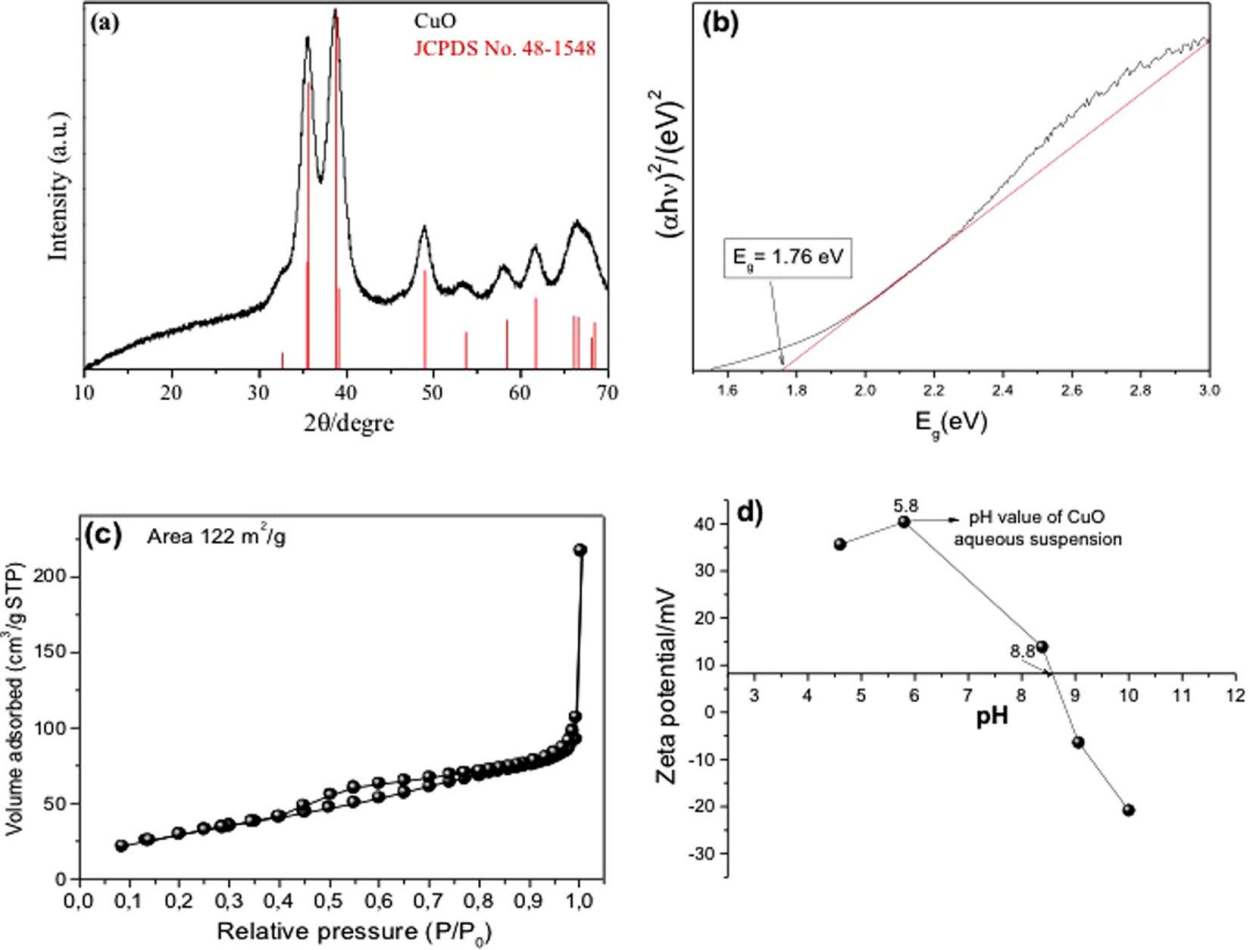
Analysis of the CuO: (**a**) XRD patterns, (**b**) UV-Vis diffuse reflectance spectra, (**c**) BET adsorption isotherm, and (**d**) zeta potential.

The XRD diffraction pattern of the CuO is shown in Fig. 1a. All the diffraction peaks could be indexed to a monoclinic structure (JCPDS 48–1548) and no impurities (such as Cu(OAc)_2_) were observed^16^. The optical characteristics of the CuO were determined by UV-visible diffuse reflectance spectroscopy (Fig. 1b). The band gaps of the CuO were determined by fitting the optical transition at the absorption edges using the Tauc model, assuming that CuO has an indirect-type transition^17^. The band gap value was obtained from the x-intecept of the tangent line for a plot of (αhν)^2^ against energy (hν), the measured band gap value was 1.76 eV^18–21^.

The porosity property of the CuO was investigated by N_2_ adsorption-desorption method (Fig. 1c). From this isotherm, it is observed that synthesized CuO nanoparticles exhibited type IV isotherm and the specific surface area was calculated by Brunauer, Emmett, and Teller (BET) method and the obtained value was 122 m^2^ g^−1^ ^22^. Zeta potential analysis was performed to determine the CuO surface charge characteristics as a function of pH, since pH exerted a strong influence on the interaction between CO_2_ and the CuO surface. Figure 1d shows the CuO zeta potential plotted as a function of pH, with a predominantly positive charge density in an aqueous medium. The suspension was at pH 6.0 without any electrolyte, and the isoelectric point of the CuO was at pH ≈ 8.8.

**Figure 2:**
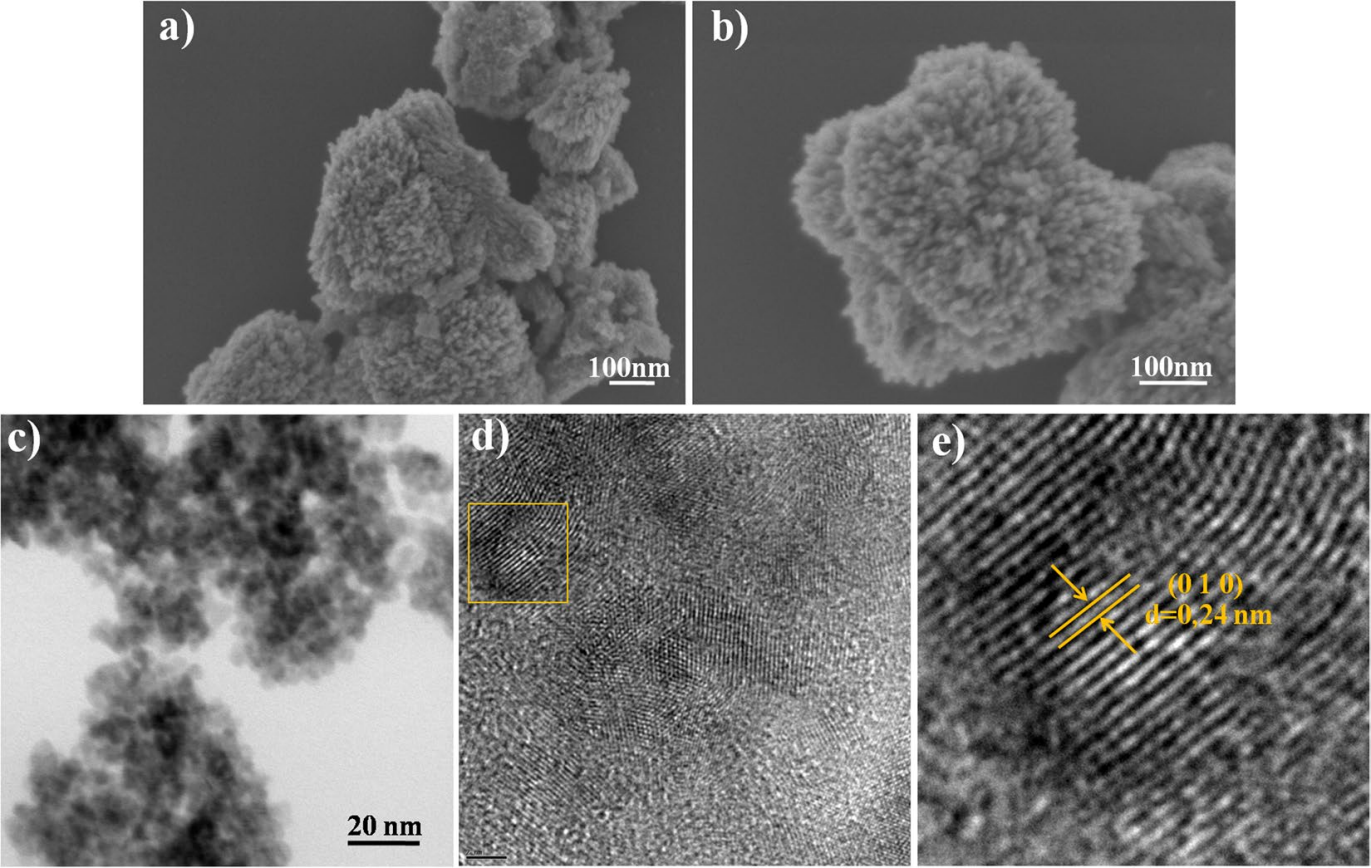
Electron microscopy analysis of CuO: (**a**,**b**) FESEM images; (**c**,**d**) low-magnification TEM images; (**e**) HRTEM image of selected area in (**d**).

The morphology of the CuO was analyzed by FESEM and HRTEM. The FESEM image (Fig. 2) revealed that the nanoparticles presented a homogeneous coral-like architecture composed of aggregates of CuO nanospheres. The HRTEM measurements (Fig. 2e) confirmed that synthesis of the CuO nanoparticles by the solvothermal method resulted in the formation of a monoclinic crystalline structure, in agreement with previous work of our group^16^.

### Photoreduction tests

#### Effect of the electrolytes

**Figure 3:**
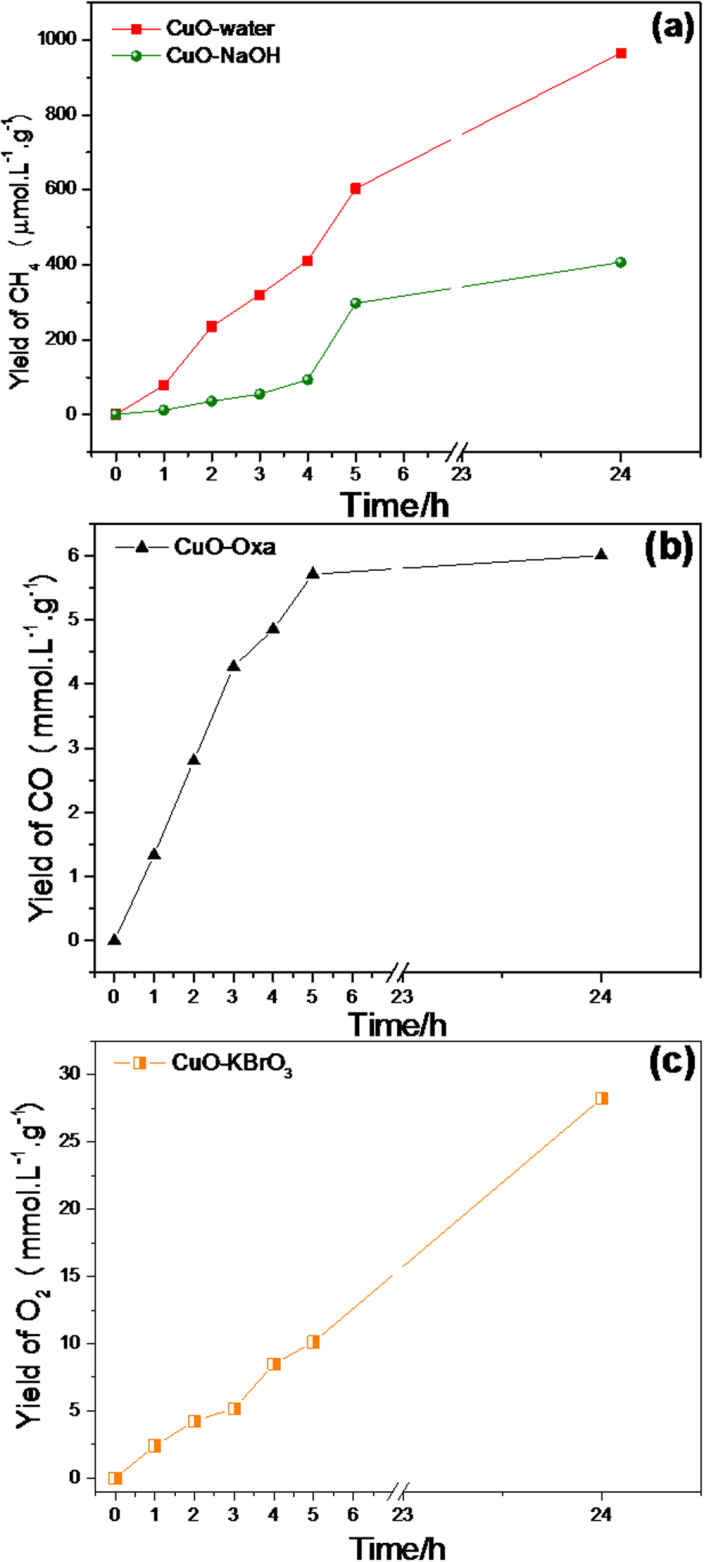
Products distribution for the photocatalytic reduction of CO_2_ with (**a**) H_2_O and NaOH, (**b**) sodium oxalate, and (**c**) KBrO_3_.

Evaluation of the photocatalytic activity of the CuO for CO_2_ photoreduction in aqueous solutions of Na_2_C_2_O_4_, KBrO_3_, and NaOH, as well as in pure water, was performed under UV irradiation (Fig. 3). Four blank condition tests were conducted in order to obtain baselines, with irradiation in the absence of the catalyst (see Supplementary Information).

Analysis of the gas samples indicated that only CH_4_ was formed when the CO_2_ photoreduction was carried out in water or in sodium hydroxide solution. Increasing formation of CH_4_ was observed during 24 h under continuous irradiation (Fig. 3a), and the results indicated that water was more effective than aqueous sodium hydroxide solution for the reduction of CO_2_ to CH_4_. This was probably related to the isoelectric point of CuO (Fig. 1d), which was at pH ≈ 8.8. Considering that the NaOH solution had pH ≈ 9–10, this indicated that the CuO surface charge was negative under this condition, with electron migration to the surface being less probable and CO_2_ adsorption not being favored. This was because at higher pH, the solubility of CO_2_ increases (forming CO_3_^2−^), hence influencing the adsorption process and interfering in the CO_2_ photoreduction^23^. However, the specie prevailing in equilibrium in our system (using other electrolytes) is HCO_3_^−^, which was assumed to be the main reactant since all reactions occurred at pH ranging from 7 to 9 in non-saline medium (in this range, at least 80% of total dissolved carbon is HCO_3_^−^)^24^.

In the first step of the photocatalytic process, CO_2_ adsorbed on the CuO catalyst surface reacted with electrons to produce carbon dioxide radicals (CO_2_^•−^), which then reacted with H^+^ to form surface CO and C, ultimately producing CH_4_ ^10,25^:

1 $${{\rm{CO}}}_{2}\mathop{\to }\limits^{{\rm{e}}-}{{\rm{CO}}}_{2}^{\bullet -}\mathop{\to }\limits^{{{\rm{H}}}^{+}+{\rm{e}}-}{\rm{CO}}\mathop{\to }\limits^{{{\rm{H}}}^{+}+2\,{\rm{e}}-}{\rm{C}}\mathop{\to }\limits^{4{{\rm{H}}}^{+}+4{\rm{e}}-}{{\rm{CH}}}_{4}$$

**Figure 4:**
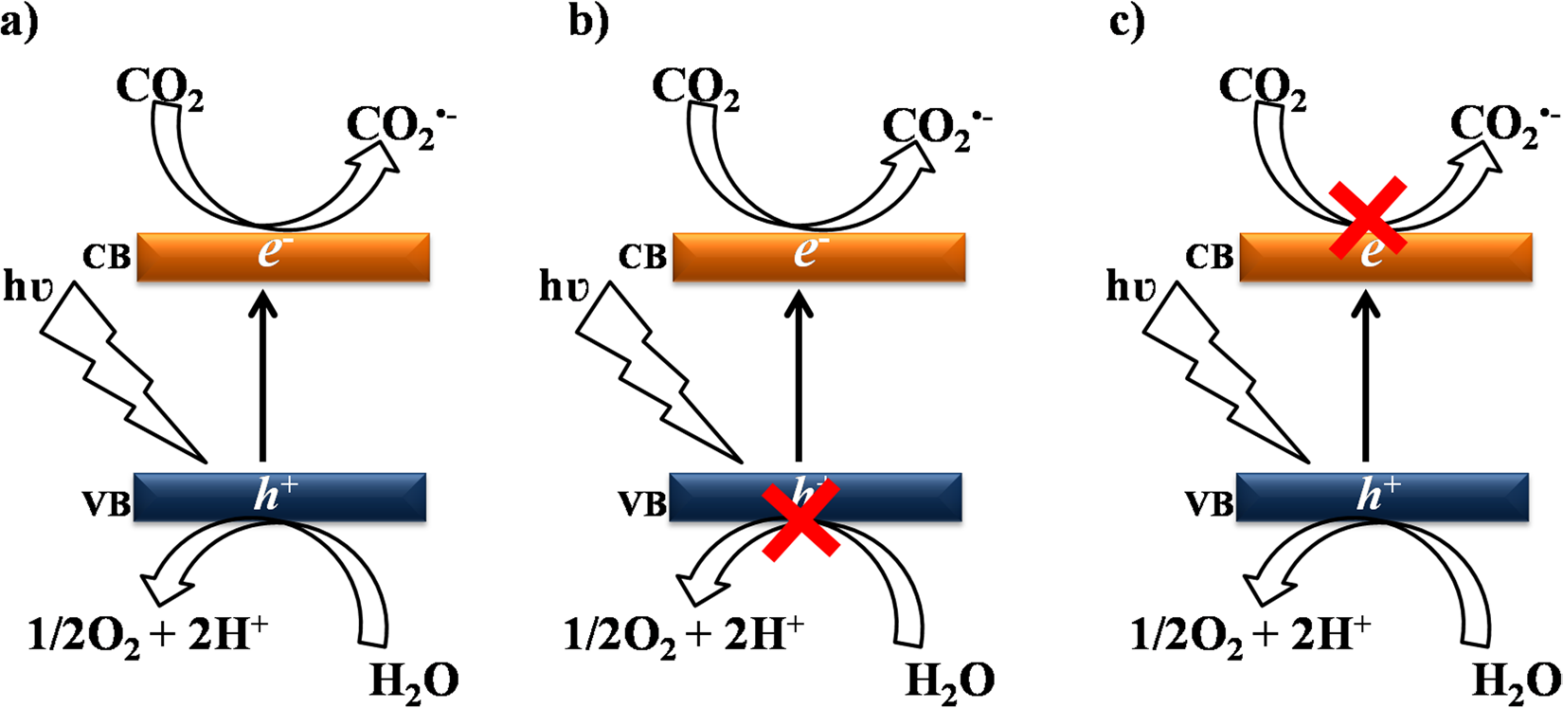
Schematic representation of the effect of the electrolyte in the photoreduction process: (**a**) pure water and NaOH, (**b**) sodium oxalate, and (**c**) KBrO_3_.

The importance of the participation of water splitting by the holes in the formation of certain products such as CH_4_ can be elucidated by the addition of species that inject electrons preferentially into the semiconductor. Sodium oxalate, for example, can be used^26^, since it reacts directly with the holes, as represented by Equation 2, so H^+^ generation is suppressed, favoring only the CO formation reaction (Equation 3) (Fig. 4b)^27,28^. However, when the reaction was carried out in aqueous KBrO_3_ solution, only O_2_ was detected, as shown in Fig. 3c. Sodium oxalate is consumed in the reaction that generates electrons, as shown in Equations 2 and 3.

2 $${({\rm{COONa}})}_{2}+2{{\rm{h}}}^{+}\to 2{{\rm{CO}}}_{2({\rm{g}})}+2{{\rm{Na}}}_{({\rm{aq}})}^{+}$$

3 $${{\rm{CO}}}_{2}^{\bullet -}+2{{\rm{CO}}}_{2}\to {\rm{CO}}+{{\rm{CO}}}_{3}^{2-}$$

In the case of O_2_ evolution, BrO_3_^−^ acts as an electron scavenger, hence suppressing any CO_2_ reduction. It is therefore expected that this compound will be reduced in the same way, forming Br^−^. The participation of electrons in the photoreduction process was related to the ability to reduce the CO_2_ present in the reaction medium to the ${{\rm{CO}}}_{2}^{\bullet -}$ radical^29^. The addition of KBrO_3_ at low concentrations impaired formation of the ${{\rm{CO}}}_{2}^{\bullet -}$radical, due to its high capacity to capture electrons. On the other hand, it hindered recombination by generating more holes for the reaction with water molecules, hence damaging the photoreduction process (Fig. 4c).

**Figure 5:**
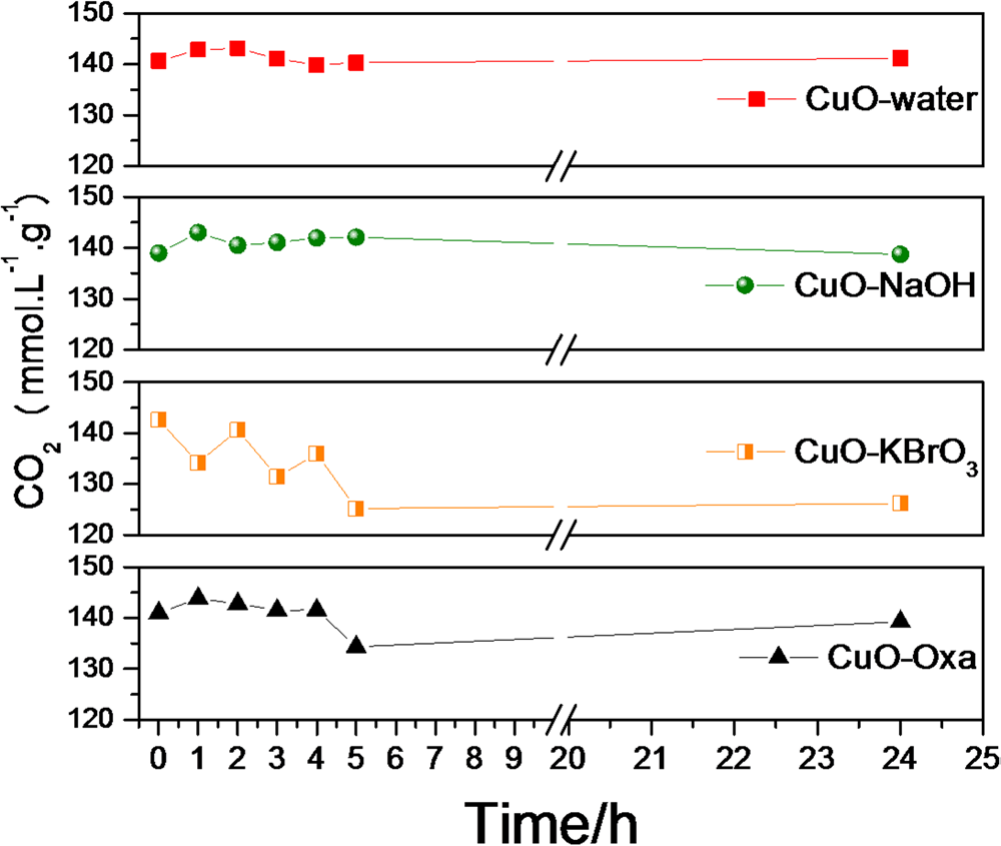
Concentration of CO_2_ as a function of UV irradiation time.

It can be seen in Fig. 5 that the amount of CO_2_ present in the headspace remained practically constant throughout the reaction (24 hours). The small oscillations observed are attributed to the displacement caused by the system in search of a chemical equilibrium between the CO_2_ dissolved in the liquid phase and that in the gas phase. The CO_2_ dissolved is consumed during the photoreaction reaction and to maintain the CO_2_ saturated medium, the gaseous CO_2_ moves into the liquid. It is worth mentioning that the long-term CO_2_ level was around 149–151 μmol.L^−1^.g^−1^ for all samples, indicating that despite the small variation observed, CO_2_ concentration could be considered constant over long periods of reaction.

#### Effect of temperature

**Figure 6:**
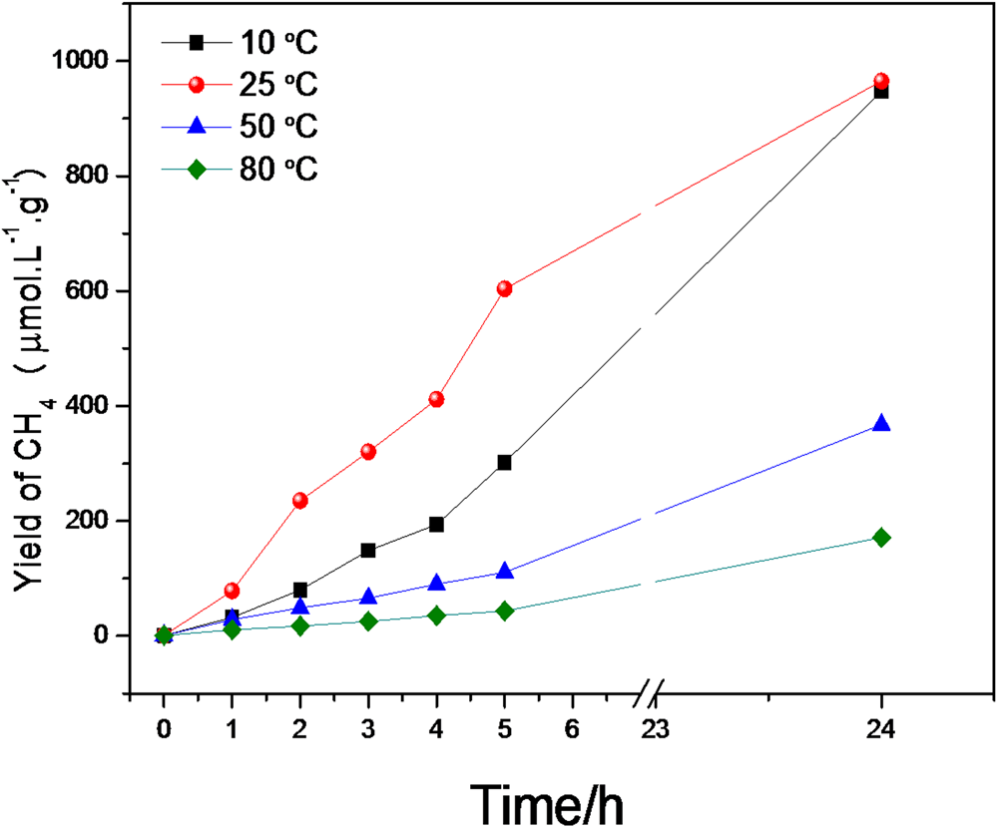
Kinetics of CO_2_ photoreduction at different temperatures.

Figure 6 shows the temperature dependence of the photoreduction of CO_2_ to CH_4_ in pure water, from which it can be seen that there was an optimum temperature for the process. This could be due to lower CO_2_ saturation (see Supplementary Material: Table S1)^30^. Therefore, very high temperatures could negatively affect the reaction rate, due to the shift of CO_2_ saturation towards lower values^31^. It can be seen from Fig. 6 that the best temperature for the photoreduction of CO_2_ to CH_4_ was around 25 ± 3 °C.

The activity was measured by the CH_4_ yields (Equation 4) and rates (Equation 5):

4 $${y}_{C{H}_{4}}=\frac{{n}_{C{H}_{2}}}{V.{m}_{cat}}$$

5 $${R}_{C{H}_{4}}=\frac{{n}_{C{H}_{4}}}{t[V.{m}_{cat}]}$$

The production rates of CH_4_ in 5 h of reaction at different temperatures are shown in Table 1. The reaction conducted at temperature of 25 °C showed the highest rate of approximately 121 μmol/L.g_cat_.h, being 13 higher than the reaction conducted at 80 °C.

**Table 1:** Yields and Rate constants for the CO_2_ photoreduction reaction catalyzed by CuO at different temperatures under UV irradiation after 5 h of reaction.

Temperature (°C)	Yield (μmol/L.g_cat_)	Rate (μmol/L.g_cat_.h^−1^)
10	299	59.8
25	606	121.2
50	110	22.0
80	44	8.8

**Figure 7:**
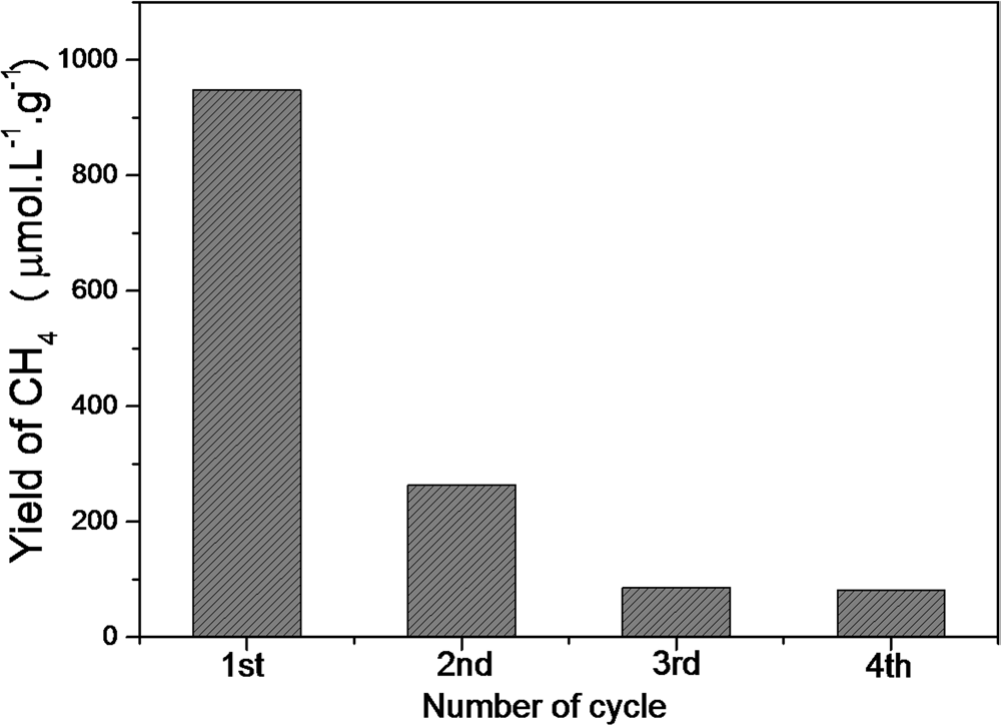
Activity of CuO after 24 h in successive cycles of the photocatalytic reduction of CO_2_ in H_2_O at 25 ± 3 °C.

The reutilization of CuO in the CO_2_ photoreduction process was evaluated in four successive runs, while keeping the experimental conditions unchanged. As shown in Fig. 7, there was a high decrease (to ≈78%) after the first photoreduction cycle, due to the conversion of CuO to copper carbonate, while the CO_2_ photoreduction became stabilized after three cycles.

All the reduction experiments (Fig. 3) showed saturation profiles, which was probably due to saturation of the headspace and consumption of the CO_2_ available for reaction (since all the experiments were performed in batch mode). However, this profile could also be related to catalyst poisoning, or to the consumption or transformation of CuO during the experiment (with the CuO acting as a reactant, rather than purely as a catalyst). In order to elucidate these possible paths, the material was characterized after the reduction reaction under each tested condition.

**Figure 8:**
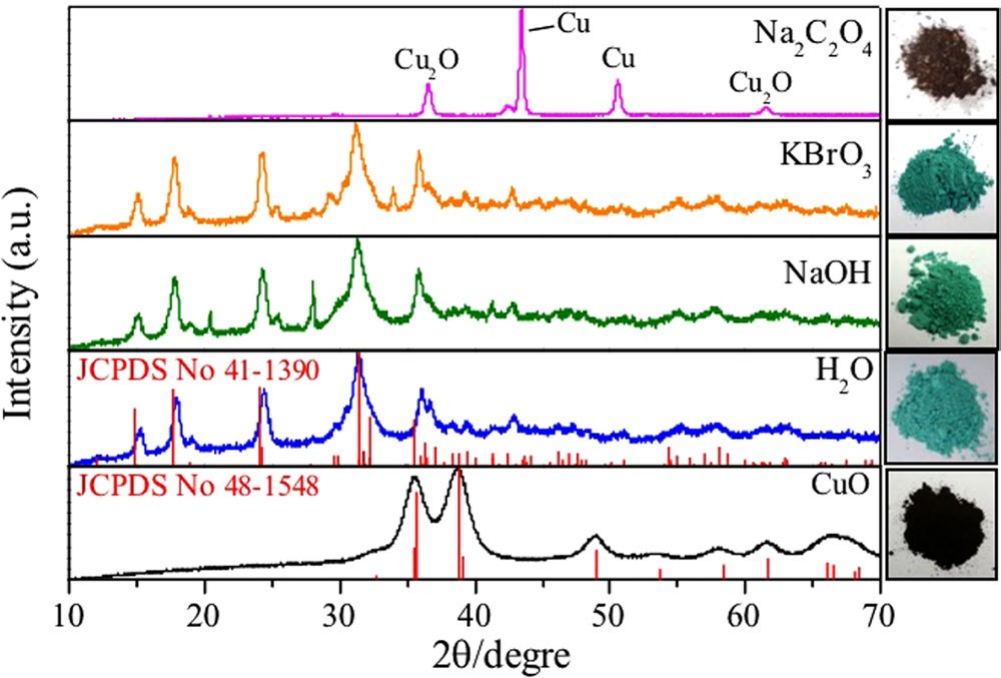
XRD patterns of catalysts before (CuO) and after reaction for 24 h under different conditions at 25 ± 3 °C.

The XRD patterns revealed noticeable changes in the structures of the materials after the reactions (Fig. 8). In the reaction using sodium oxalate solution, the material presented 2θ peaks at 43.5° and 50.5°, corresponding to planes (111) and (200), respectively, related to metallic copper (JCPDS 04-0836), together with peaks centered at 2θ of 36.5° and 61.0°, related to Cu_2_O, showing that after 24 h the material had undergone a reduction process. On the other hand, when the reaction was performed using KBrO_3_ or NaOH solutions, or pure water, the materials showed diffraction peaks related to copper carbonate (malachite, CuCO_3_.Cu(OH)_2_) (JCPDS 01-0959). In fact, the first evidence of reaction of CO_2_ with CuO was a color change from brown to light green, indicative of copper carbonate formation^32,33^:

6 $$2{{\rm{Cu}}}_{({\rm{s}})}+{{\rm{H}}}_{2}{{\rm{O}}}_{({\rm{l}})}+{{\rm{CO}}}_{2({\rm{g}})}+{{\rm{O}}}_{2({\rm{g}})}\to {\rm{Cu}}{({\rm{OH}})}_{2({\rm{s}})}+{{\rm{CuCO}}}_{3({\rm{s}})}$$

**Figure 9:**
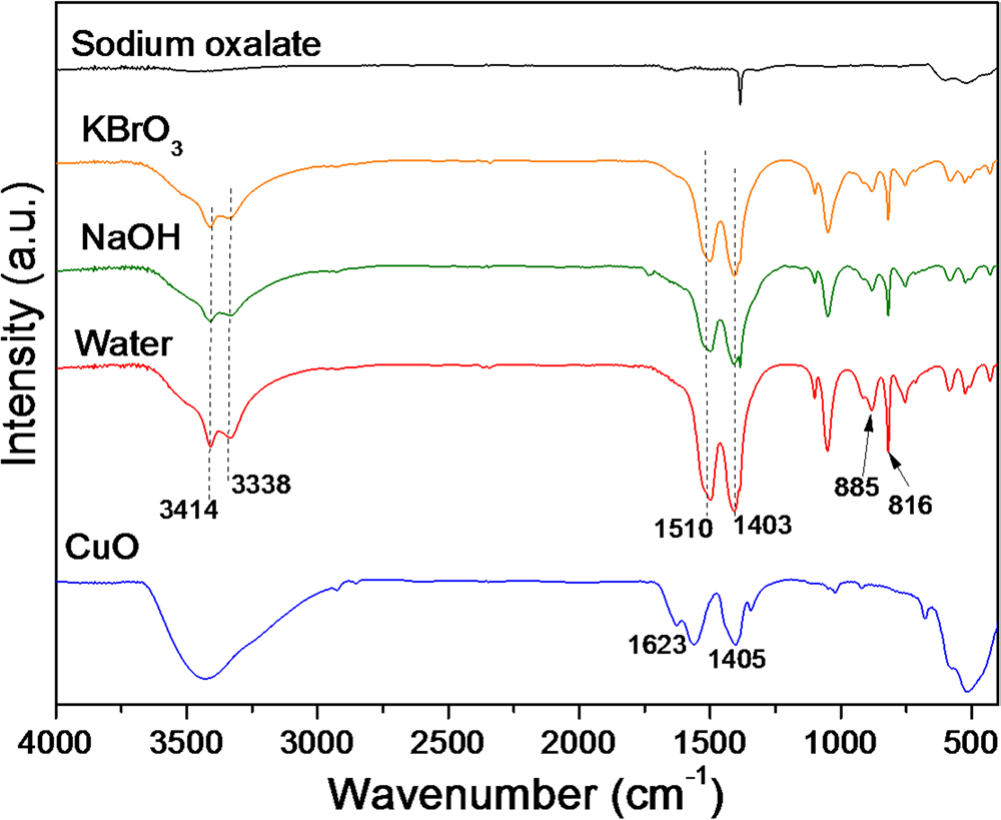
FTIR spectra of the materials after CO_2_ photoreduction.

The FTIR spectra of the CuO surface before and after UV irradiation for 24 h are shown in Fig. 9. The FTIR spectrum of the as-prepared CuO showed a broad band at approximately 400–600 cm^−1^, attributed to the vibrations of Cu-O, and bands at 1623 and 1405 cm^−1^, related to asymmetric stretching of C-O and asymmetric bending of CH_3_ of the copper acetate precursor, respectively^23^. After the CO_2_ photoreduction using different electrolytes the FTIR spectra showed a different profile of the CuO sample before the reaction. Bands at 1510 cm^−1^ and 1403 cm^−1^ were related to C-O stretching modes, while those at 885 and 816 cm^−1^ were due to C-O bending vibration modes. Bands at 3414 and 3338 cm^−1^ could be attributed to O-H stretching modes, reflecting the presence of two different OH groups in the copper carbonate crystal lattice^34–36^.

**Figure 10:**
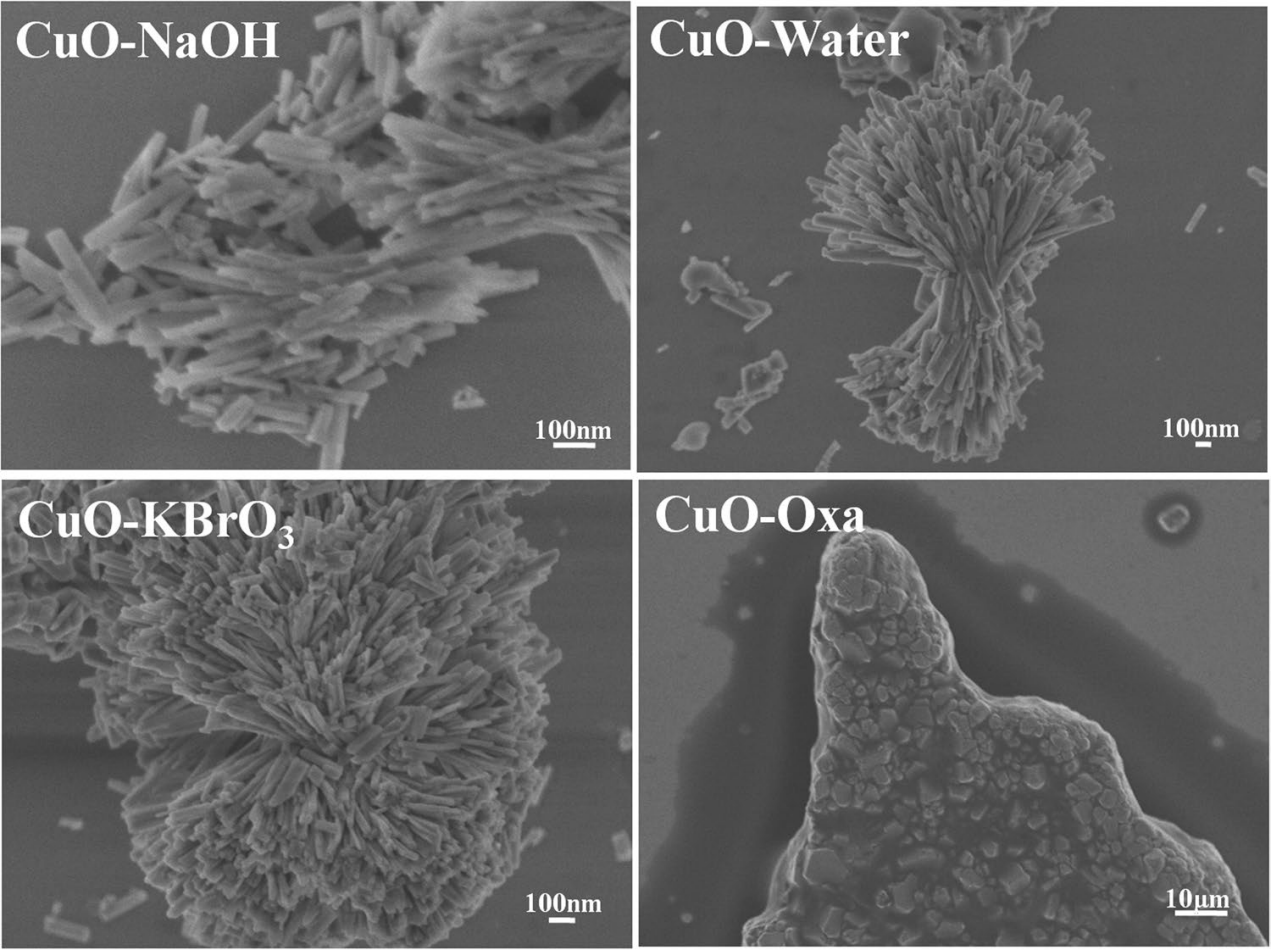
FESEM images of the materials after 24 h of reaction.

**Figure 11:**
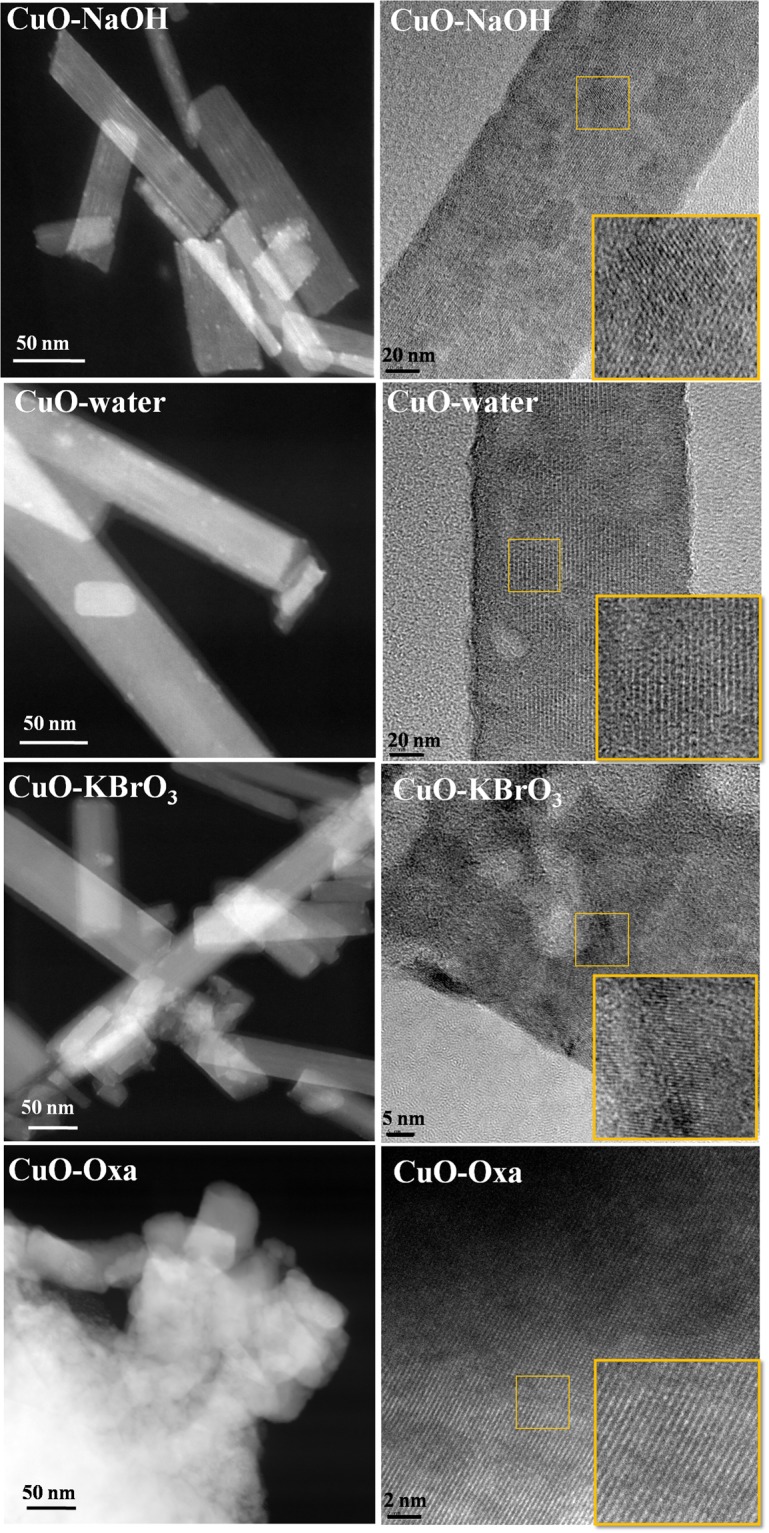
HRTEM images of the materials after 24 h of reaction.

FESEM and HRTEM was employed to examine morphological features of the materials after the photoreduction process (Figs 10 and 11). It was observed that not only did the CuO structure change, but the morphology also altered. The reactions performed in the presence of pure water and aqueous solutions of NaOH or KBrO_3_ led to the formation of nanorods, while the reaction carried out in the presence of aqueous sodium oxalate solution led to the formation of metallic copper plates.

The findings were supported by comparison with conversion levels reported in the literature (Table 2). Considering studies with copper oxide and CuO-related materials, stable CO_2_ conversions catalyzed by malachite were within the same range, suggesting that the same phenomenon was probably being observed (despite the fact that in all the earlier studies it was stated that the actual catalyst present was copper oxide).

**Table 2:** CuO-based photocatalytic systems for CO_2_ reduction to CH_4_.

Catalysts	Condition	Irradiance	CH_4_ (μmol.g^−1^ h^−1^)	Ref.
CuO	300 W Xe lamp	100 mW cm^−2^	2.96	^9^*
CuO	100 W Xenon solar	—	19.7	^10^*
Cu_2_O	125 W Hg lamp	—	16.0	^11^
Cu_2_O/TiO_2_	300 W Xe lamp	20.5 mW cm^−2^	0.99	^12^
CuO	5 W UVC lamp	5.5 mW cm^−2^	39.5	This work

^*^Normalized units.

**Figure 12:**
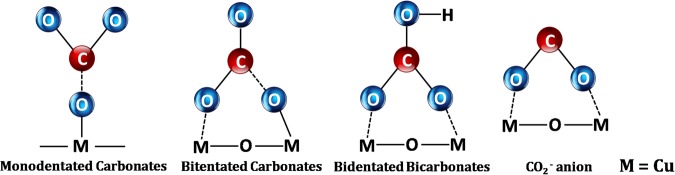
Schematic diagram of species adsorbed on the CuO surface in the CO_2_ adsorption process.

From these results, we propose that the actual catalyst for CO_2_ reduction was the as-formed malachite phase, acting by surface adsorption and possible structural exchange. CO_2_ could be bonded to the surface of the material in three different ways, as monodentate carbonate, bidentate carbonate, and bidentate bicarbonate, as shown in Fig. 12 ^4^. Under UV irradiation, the species adsorbed on the material surface could be easily transformed to CO_2_^•−^, which is a key intermediate in CO_2_ photoreduction.

## Methods

### Solvothermal synthesis of CuO

The CuO nanoparticles were synthesized by adding 50 mL of a 0.05 M solution of copper acetate in ethanol (99.5%, Synth) to a 100 mL poly(tetrachlorethylene) capsule and then placing the capsule in an autoclave, under constant magnetic stirring^37^. The solvothermal treatment was performed at 110 °C for 20 h. After the reaction, the autoclave was cooled naturally to room temperature. The product was recovered by centrifugation, washed two times in ethanol, and then dried in air at 60 °C for 4 h.

### Characterization

The CuO powder was characterized by X-ray diffraction (XRD), using CuKα radiation with λ = 0.15406 nm, employing a Shimadzu XRD 6000 diffractometer operated at 30 mA and 30 kV, in the 2θ range from 20 to 80°, with a scan step of 0.02°. The morphologies of the materials were characterized by high resolution transmission electron microscopy (HRTEM), using a TECNAI G2 F20 microscope (FEI) operated at 200 kV, and by field emission scanning electron microscopy (FESEM), using a JSM 6701 F microscope (JEOL) operated at 5 kV. The HRTEM samples were prepared by wetting carbon-coated copper grids with a drop of the colloidal suspensions and then drying in air.

The specific surface areas of the materials were measured using nitrogen adsorption at 77 K (ASAP-2020, Micromeritics), with calculation according to the Brunauer-Emmett-Teller (BET) method. Prior to the analyses, the samples were pre-treated (degassed) by heating at 70 °C under vacuum until reaching a pressure of less than 20 mm Hg. Diffuse reflectance spectra (DRS) in the ultraviolet-visible region were recorded between 200 and 800 nm, at room temperature, using a Cary 5 G instrument (Varian) operated in diffuse reflectance mode. The band gaps of the samples were determined according to the method proposed by Tauc^38,39^. Infrared (FTIR) spectra of the materials were obtained in the range from 4000 to 400 cm^−1^, with 32 scans and 4 cm^−1^ resolution, using a Spectrum 1000 spectrophotometer (Perkin Elmer). The zeta potentials of dilute suspensions of the materials were measured with a Zeta Sizer nano-ZS instrument (Malvern Instruments), in the pH range from 11 to 4, with the pH adjusted by adding 0.1 M HCl or 0.1 M NaOH.

### CO_2_ photoreduction

The CO_2_ photoreduction was performed in a 500 mL capacity cylindrical acrylic reactor, covered with borosilicate glass. A 0.3 g quantity of the catalyst was suspended in 300 mL of solutions of NaOH (0.1 M), Na_2_C_2_O_4_ (0.1 M), or KBrO_3_ (0.1 M), or in pure water. Ultrapure CO_2_ was bubbled through the reactor for at least 20 min to ensure that all the dissolved oxygen was eliminated. The illumination system employed a 5 W UVC lamp (Philips) with a wavelength of 253.7 nm, positioned in the center of the reactor. The measured intensity of the incident light was 5.5 mW cm^−2^. A detailed description of the photoreactor system is provided in the Supplementary Information.

The progress of the reaction was monitored by collecting and analyzing samples at regular intervals. Gaseous products were determined by GC-TCD and GC-FID (model CP-3800 gas chromatograph, Varian), using a packed column (HayeSep N, 0.5 m × 1/8″). The gas flow rates were 30 mL min^−1^ (H_2_), 300 mL min^−1^ (air), and 30 mL min^−1^ (N_2_). The injector, TCD, and FID temperatures were 150, 200, and 150 °C, respectively. The sample injection volume was 2 μL, and the yield was calculated using injections of standard gaseous mixtures. Blank reactions were carried out to ensure that the CH_4_ and CO originated from the photoreduction of CO_2_ see Supplementary Information. In the first blank reactions, no catalyst was added, and all other conditions were maintained the same, in the second test the reaction was conducted in nitrogen atmosphere.

## Conclusion

Concluding, we observed that CuO was not stable during the CO_2_ photoreduction process, with CuO changing to malachite (CuCO_3_.Cu(OH)_2_). However, significant CO_2_ conversion was observed during the CuO carbonation process, and the performance of malachite as a catalyst was comparable to results reported in the literature, where the catalyst was assumed to be CuO. The nature of the electrolyte influenced product selectivity, with CuO phase change participating in the processes. The results reported here contribute to elucidation of the role of CuO in the CO_2_ photoreduction process, providing important information for the rational development of Cu-based catalysts for this process.

## Electronic supplementary material

Supplementary Information
