# Supplementary material for: Insights into the role of CuO in the CO2 photoreduction process
Source: Sci Rep. 2019 Feb 4;9:1316. doi: 10.1038/s41598-018-36683-8 (PMC6361925; doi:10.1038/s41598-018-36683-8)
Supplement: Supplementary file 1 — Supplementary Information [file 41598_2018_36683_MOESM1_ESM.docx]

**Insights into the role of CuO in the CO_2_ photoreduction process**

André E. Nogueira^a,b^, Jéssica A. Oliveira^b,c^, Gelson T. S. T. da Silva ^b,d^, Caue Ribeiro^b*^

*^a^Brazilian Nanotechnology National Laboratory (LNNano), Brazilian Center for Research in Energy and Materials (CNPEM), Zip Code 13083-970, Campinas, São Paulo, Brazil.*

*^b^Embrapa Instrumentation, Rua XV de Novembro, 1452, CEP: 13560-970, CP 741, São Carlos, SP, Brazil.*

*^c^Department of Chemical Engineering – Federal University of São Carlos, Via Washington Luiz, km 235, CEP: 13565-905, São Carlos, SP, Brazil.*

*^d^Department of Chemistry – Federal University of São Carlos, Via Washington Luiz, km 235, CEP: 13565-905, São Carlos, SP, Brazil*

* Corresponding author: caue.ribeiro@embrapa.com.br

Tel.: +55 16 2107 2800; fax: +55 16 2107 2902


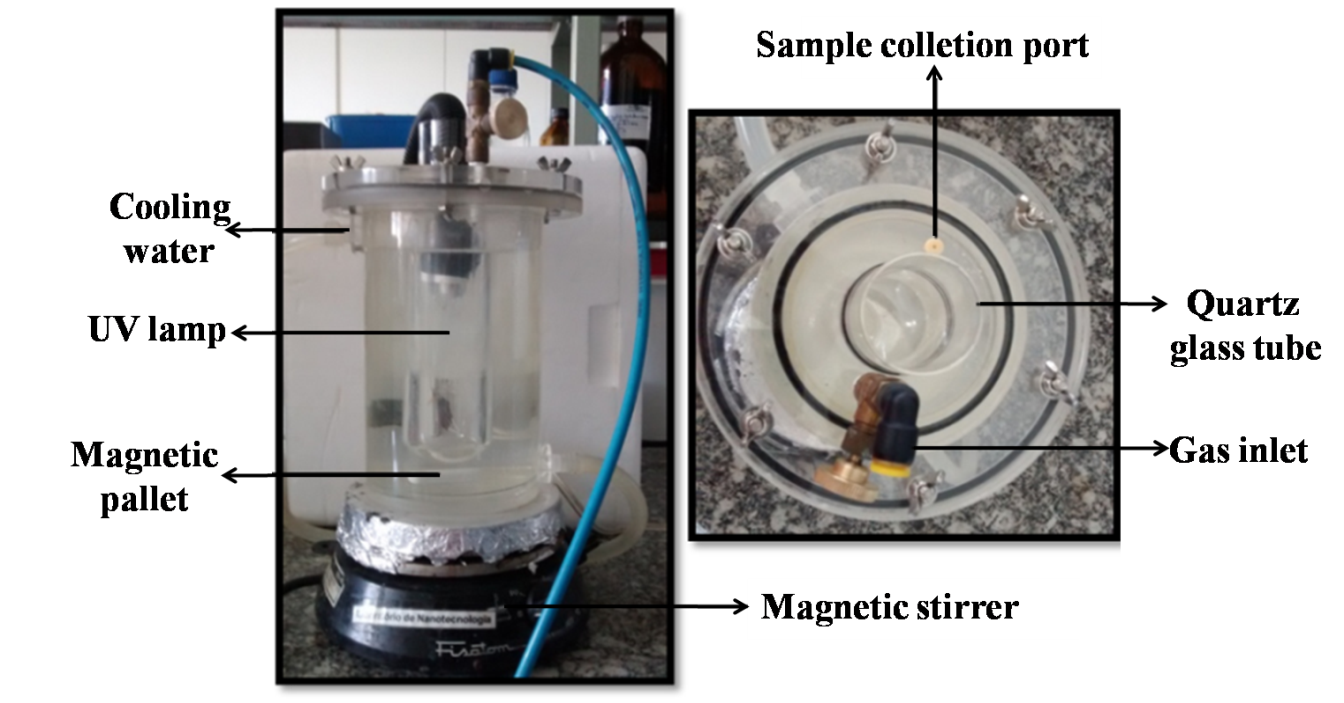


**Fig. S1.** Photocatalytic reactor system.

**
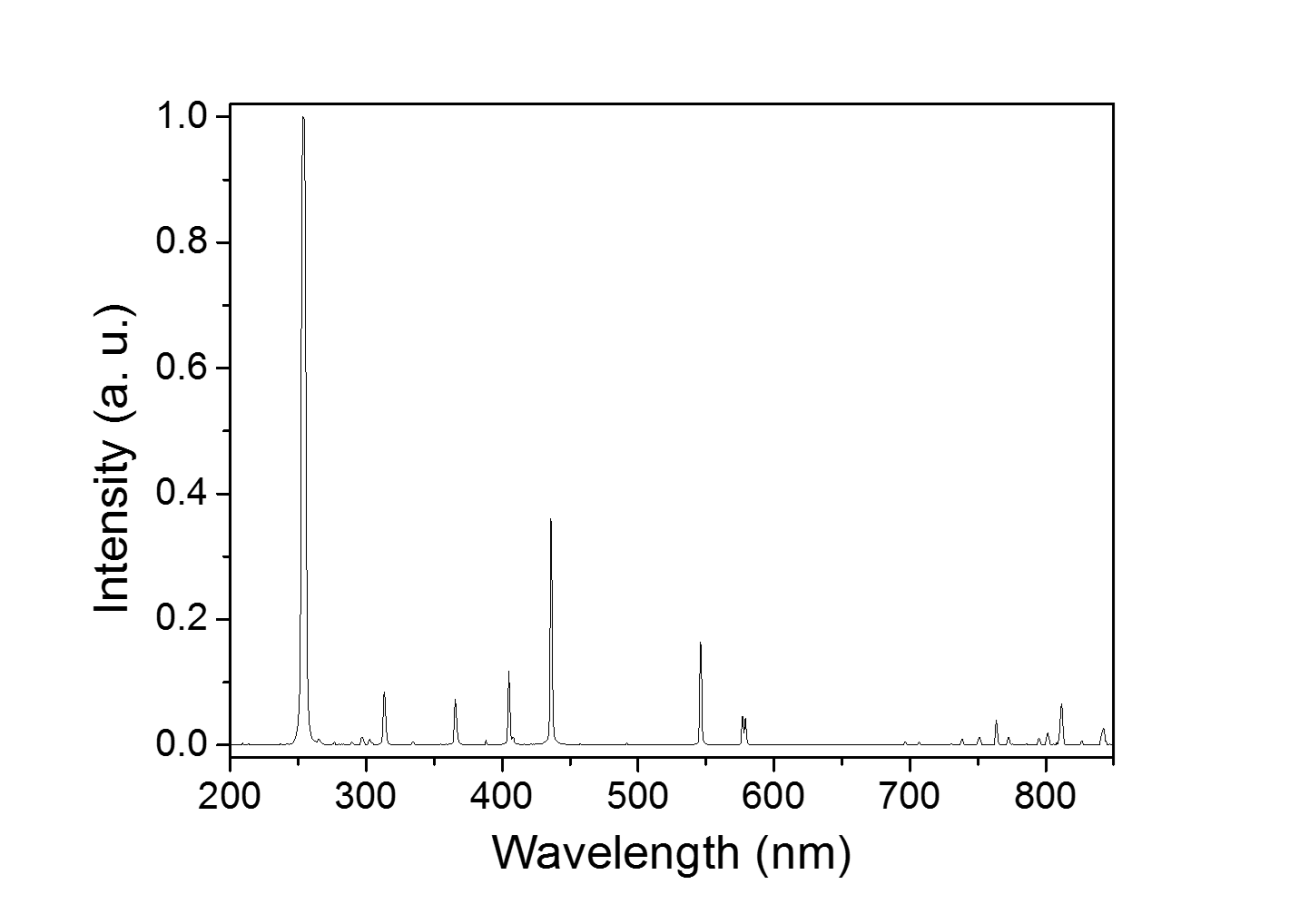
**

**Fig S2.** Light spectrum of the UVC (5W) lamp.


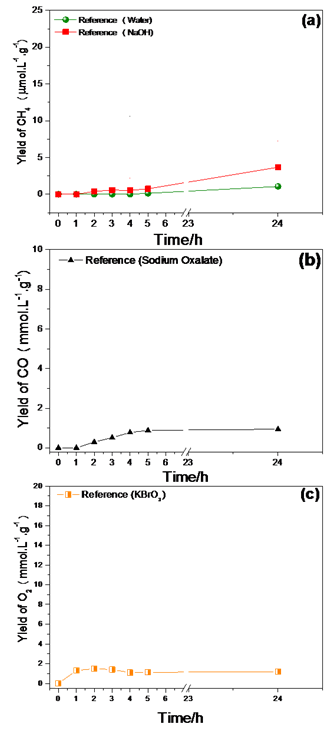


**Fig. S3**. CO_2_ photoreductionwithout semiconductor under UV radiation at 25±3 °C.

**Fig. S4.** UV-vis diffuse reflectance spectra (DRS) of CuO.

**Table S1:** Solubilities for carbon dioxide in water at atmospheric pressure and at different temperatures.

| T(ºC) | CO­_2_ solubility (mmol/atm) |
| --- | --- |
| 10 | 0.962 |
| 25 | 0.609 |
| 50 | 0.319 |
| 80 | 0.127 |
